# Deciphering of a Putative GPER Recognition Domain in ERα and ERα36

**DOI:** 10.3389/fendo.2022.943343

**Published:** 2022-06-30

**Authors:** Alexandre Acramel, Yves Jacquot

**Affiliations:** ^1^ CiTCoM laboratory, Centre National de la Recherche Scientifique (CNRS) Unité Mixte de Recherche (UMR) 8038, Institut National de la Santé et de la Recherche Médicale (INSERM) U1268, Faculty of Pharmacy of Paris, Université Paris Cité, Paris, France; ^2^ Department of Pharmacy, Institut Curie, Paris, France

**Keywords:** GPER, ERα, ERα36, binding domain, breast cancer

## Introduction

Twenty-five years ago, a class A (rhodopsin-like) G protein-coupled receptor (GPCR) was reported to participate in the rapid physiological responses to the mammalian steroid hormone 17β-estradiol (E2). Initially found in human B cells (1), it was further identified in breast carcinoma cells (2). As no endogenous ligand was identified, this membrane receptor was called GPR30, following the numbering system of orphan receptors. In 2005, two independent groups demonstrated an E2 interaction with GPR30 characterized by a Kd value in the nanomolar range (3, 4). Thus, it was renamed GPER for G protein-coupled estrogen receptor. The GPER structure differing strongly from the canonical estrogen receptors ERα and β and the 46 kDa ERα truncated isoform (ERα46), impassioned debates focusing on its intracellular localization, its exact role regarding nuclear and membrane signaling pathways and its physical interaction with ERα were carried out (5, 6). Shortly afterwards, a direct interaction of GPER with activated ERα and the epidermal growth factor receptor (EGFR), which both support transactivation, was evidenced through biochemical methods (7–9).

GPER participating in breast cancer development including triple negative breast cancer (TNBC) and in tamoxifen resistance (10–12), it would be interesting to define the ERα region involved in ERα/GPER interaction. Such a finding could lead to innovative therapeutic strategies targeting aggressive breast tumors. Based on recently published data, we report here a putative GPER-binding domain located in the hinge region of ERα and also found in the 36 kDa ERα truncated isoform (ERα36).

## Deciphering of an ERα and ERα36 Platform Putatively Involved in GPER Interaction

### ERα and ERα36 Sequence Alignment

**Figure 1:**
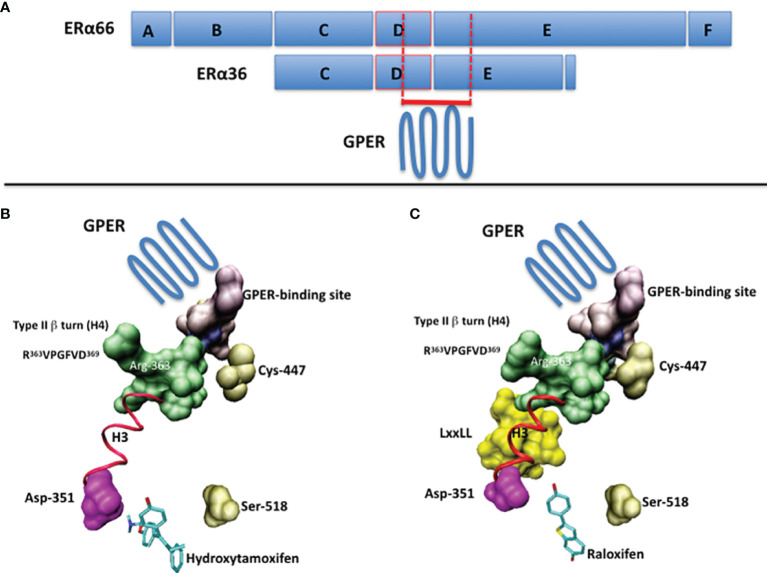
**(A)** The full-length ERα (ERα66) and the ERα36 isoform are schematized by boxes. The A/B domains contain the transactivation function AF1, the C domain corresponds to the DNA-binding domain (DBD), the D domain corresponds to the hinge region and the E/F domains contain the ligand-binding domain (LBD) and the transactivation function AF2. The putative GPER-binding site overlaps the C-terminus of the D domain and the N-terminus of the E/F domains. **(B)** The suspected GPER-binding domain in ERα, according to ERα/ligand crystal structures, in the presence of 4-hydroxytamoxifen (PDB code: 1ERT) and **(C)** raloxifen (PDB code: 1GWQ). Ligands are drawn in cyan. The part of the 295-311 sequence available in PDB structures is in purple. In pink, the aspartic acid 351, with which interacts the basic chain of 4-hydroxytamoxifen. In gold, the cystein 447, which is palmitoylated prior to membrane targeting, and the serine 518, which participates in the stabilization of the ligand within the ligand-binding pocket. In green, the side chain of the Arg-363 of the type II β-turn (R^363^VPGFVD^369^). In yellow, the coactivatory motif LxxLL. The regions of interest are visualized by using Connolly surface. In red (ribbon): the helix H3. Drawings have been performed on a Silicon Graphics O2 workstation using the Insight II software package (version 98.0, Accelrys, Inc, San Diego). Due to the presence of a proteolytic site in the 295-311 sequence of ERα, only the region 305-311 is available.

The ERα and ERα36 primary structures contain the short sequence PLMIKRSKKNSLALSLT, which corresponds to the residues 295-311 and 123-139, respectively (13, 14). In the context of ERα, this sequence is not only targeted by post-translational modifications, but is also partially structured into polyproline II, a conformational state usually found in interaction modules (14). Moreover, it overhangs a type II β turn (sequence RVPGFVD, residues 363-369, helix H4 of ERα), whose orientation depends strongly on the bound ligand, as shown in **Figure 1** (14). Accordingly, it binds the ubiquitin ligase E6AP (15) and Ca^2+^-calmodulin (16). Thus, this region is important for the recruitment of proteins and the control of transcription.

### Identification From the ERα and ERα36 Primary Sequences of the First Peptidic GPER Modulator

The peptide ERα17p, which corresponds to the aforementioned 295-311 and 123-139 fragments of ERα and ERα36, respectively, interacts with Ca^2+^-calmodulin (17), Hsp70 (18) and ERα, itself (19). As its antiproliferative activity is observed not only in ERα-positive but also in ERα-negative breast cancer cells, a role of GPER should be suspected (20, 21). By using a fluorescein-labeled version of ERα17p combined with a specific GPER immunohistochemical staining approach, a co-localization has been shown at the cytoplasmic membrane, suggesting a direct interaction between ERα17p and GPER (22). Following *in silico* studies, the ERα17p sequence seems to interact with the same GPER binding site as conventional ligands (22). The role of GPER in the mechanism of action of ERα17p is confirmed by the inhibition of the ERα17p-mediated antiproliferative action by the selective GPER antagonist G-36 (22). ERα17p behaves as a GPER inverse agonist, as usually found with intrinsically activated GPCRs. It induces a proteasome-dependent decrease of GPER, which is followed by a decrease of pEGFR, pERK1/2 and of the amount of c-fos (22). Finally, we have demonstrated that G-15, another selective GPER antagonist, was able to inhibit the anti-hyperalgesic and anti-inflammatory actions induced by ERα17p, *in vivo* (23).

Altogether, these results reveal not only that the 295-311 and 123-139 regions of ERα and ERα36, respectively, interfere with GPER, but also that these two regions could constitute a putative GPER-interacting platform.

## Discussion

Additionally to EGFR, a direct interaction between GPER and ERα has been evidenced by using co-immunoprecipitation (8). As this interaction is enhanced by E2 and prevented by fulvestrant (ICI 182,780), a ligand-dependent process seems likely. Fulvestrant being a GPER agonist (7) and an ERα antagonists (24), a mechanism depending on ERα should be stressed. By using immunohistochemistry in human primary monocytes expressing ERα36, an E2-independent physical interaction between ERα36 and GPER has been evidenced (25). The absence of E2 effects may result, in this context, from the lack of AF2 domain in ERα36 and, consequently, from a lack of E2 interaction (9, 26). However and as E2 participates in ERα36 signaling, the involvement of GPER as an alternative E2-interacting target is not excluded.

In connection with previous results, we have attempted to identify a GPER-binding surface in ERα and ERα36. The approach consisting in exploring interaction patterns of a pharmacologically active peptide issued from a disordered or folded protein domain is often linked to protein-protein recognition (27, 28). Accordingly, data reported above with the peptide ERα17p suggest the presence of 1) a putative GPER interaction module in both, ERα (residues 295 to 311) and ERα36 (residues 123 to 139), and 2) structural elements in the full-length ERα, only, that would be required for the ligand-dependency of this interaction. As ERα36 fails to interact with E2 (26), the association of GPER with the ERα36 residues 123-139 may explain how E2 participates, albeit indirectly, in the ERα36-dependent transcriptional machinery, and why tamoxifen is mitogenic in ERα-negative breast cancer cells (29). As such and depending on the physiopathological context, the cellular phenotype (expression levels of ERα, ERα36 and EGFR) and the level of E2, the protein GPER can be considered either as an ERα coactivator or as an E2-binding protein, *per se* (9). In this regard, the side chain of the arginine 363, which is located in the type II β-turn (helix H4, residues 363-369 of ERα) and on which overhangs the 295-311 sequence, may play a role as its orientation varies in a ligand-dependent manner to allow or not the recruitment of coactivators, as exemplified with tamoxifen and raloxifene (**Figure 1**). This observation could also be linked to the high flexibility of this surface-exposed region (14, 19). Hence, the recruitment of GPER by ERα appears highly dependent to the hormonal context and the conformational state of ERα, at the atom scale.

Targeted mutagenesis in the putative GPER-binding region of ERα and ERα36 could allow better understanding of the direct interaction between GPER and ERα as well as ERα36 (30). GPER X-ray structure could also be helpful in this respect. Lastly, our model could contribute to the development of new GPER modulators and, therefore, of new clinical approaches, in the context of breast cancer including TNBC.

## Author Contributions

YJ has designed and intellectually contributed to this work. AA has participated to the writing of this article. YJ and AA approved it for publication.

## Conflict of Interest

The authors declare that the research was conducted in the absence of any commercial or financial relationships that could be construed as a potential conflict of interest.

## Publisher’s Note

All claims expressed in this article are solely those of the authors and do not necessarily represent those of their affiliated organizations, or those of the publisher, the editors and the reviewers. Any product that may be evaluated in this article, or claim that may be made by its manufacturer, is not guaranteed or endorsed by the publisher.
